# Parvovirus B19 infection and severe anaemia in Kenyan children: a retrospective case control study

**DOI:** 10.1186/1471-2334-10-88

**Published:** 2010-04-03

**Authors:** James Wildig, Yvonne Cossart, Norbert Peshu, Nimmo Gicheru, James Tuju, Thomas N Williams, Charles R Newton

**Affiliations:** 1 Department of Infectious Diseases and Immunology, University of Sydney, Australia; 2 Centre for Geographical Medicine, KEMRI-Wellcome Trust, Kenya Medical Research Institute, Kilifi, Kenya; 3 Nuffield Department of Clinical Medicine, John Radcliffe Hospital, Oxford OX3 9DU, UK; 4 Clinical Research Unit, London School of Hygiene and Tropical Medicine, UK; 5 Institute of Child Health, University College London, UK

## Abstract

**Background:**

During acute *Human parvovirus B19 *(B19) infection a transient reduction in blood haemoglobin concentration is induced, due to a 5-7 day cessation of red cell production. This can precipitate severe anaemia in subjects with a range of pre-existing conditions. Of the disease markers that occur during B19 infection, high IgM levels occur closest in time to the maximum reduction in haemoglobin concentration. Previous studies of the contribution of B19 to severe anaemia among young children in Africa have yielded varied results. This retrospective case/control study seeks to ascertain the proportion of severe anaemia cases precipitated by B19 among young children admitted to a Kenyan district hospital.

**Methods:**

Archival blood samples from 264 children under 6 years with severe anaemia admitted to a Kenyan District Hospital, between 1999 and 2004, and 264 matched controls, were tested for B19 IgM by Enzyme Immunosorbent Assay and 198 of these pairs were tested for B19 DNA by PCR. 536 samples were also tested for the presence of B19 IgG.

**Results:**

7 (2.7%) cases and 0 (0%) controls had high B19 IgM levels (Optical Density > 5 × cut-off value) (McNemar's exact test p = 0.01563), indicating a significant association with severe anaemia. The majority of strongly IgM positive cases occurred in 2003.

10/264 (3.7%) cases compared to 5/264 (1.9%) controls tested positive for B19 IgM. This difference was not statistically significant, odds ratio (OR) = 2.00 (CI_95 _[0.62, 6.06], McNemar's exact test p = 0.3018. There was no significant difference between cases and controls in the B19 IgG (35 (14.8%) vs 32 (13.6%)), OR = 1.103 (CI_95 _[0.66, 1.89], McNemar's exact test, p = 0.7982), or the detection of the B19 DNA (6 (3.0%) vs 5 (2.5%)), OR = 1.2 (CI_95 _[0.33, 4.01], McNemar's exact test p = 1).

**Conclusions:**

High B19 IgM levels were significantly associated with severe anaemia, being found only among the cases. This suggests that 7/264 (2.7%) of cases of severe anaemia in the population of children admitted to KDH were precipitated by B19. While this is a relatively small proportion, this has to be evaluated in the light of the IgG data that shows that less than 15% of children in the study were exposed to B19, a figure much lower than reported in other tropical areas.

## Background

Severe anaemia (haemoglobin less than 50 g/l) is a major cause of death among young children in malaria endemic areas, including sub-Saharan Africa [1-3]. In such areas the majority of children have mild to moderate anaemia arising from factors which reduce red cell survival time and/or inhibit erythropoiesis, including *P falciparum *infection, iron deficiency, protein-energy malnutrition, haemoglobinopathies and other infections including HIV [4]. Parvovirus B19 (B19), which is common throughout the world, has the potential to precipitate severe anaemia in children with pre-existing moderate anaemia, particularly those with significant haemolysis. The acute infection causes cessation of erythropoiesis for 5-7 days arising from apoptosis of red cell precursors in the bone marrow, which decreases the haemoglobin level for 1-2 weeks until bone marrow recovery [5]. After contact with an infectious dose of the virus, rapid viral replication in erythroid progenitor cells results in a high level viraemia which is terminated by the production of specific IgM antibody beginning around day 9. A number of studies have described high levels of IgM lasting for 1-2 weeks (coinciding with the time of haemoglobin reduction) [6,7]. Lower levels of IgM usually persist for 2-3 months or more. After the high initial viral load lower levels of B19 DNA can often be detected by nucleic acid amplification techniques for 6 months or more, although in other cases viral DNA drops below detectable levels within weeks [8]. Thus, of the markers of B19 infection, high IgM concentrations are most closely associated in time with the period of reduced haemoglobin.

Recently B19 infection was found to be significantly associated with severe anaemia among young children living in a malaria-endemic region of Papua New Guinea (PNG) [9]. Previous studies of the contribution of B19 to severe anaemia among young children in Africa have yielded varied results [2,3,10,11]. Here we present the results of a retrospective case-control study investigating the association between parvovirus B19 and severe anaemia in a malaria-endemic area of Kenya.

## Methods

This retrospective case/control study used archived blood samples stored at -80°C that had been collected from all children admitted to Kilifi District Hospital, Kenya between January 1999 and December 2004. Cases were defined as children ≤ 6 years of age with a haemoglobin concentration ≤ 50 g/l, and 50 were selected randomly from each year 1999 to 2004. Controls with haemoglobin > 50 g/l matched for age (+/- 6 months), sex and date of admission (+/- 1 month) were randomly selected from the hospital admissions (Table 1). A person unaware of the clinical status performed the assays.

**Table 1:** Demographic characteristics of case/control population

	Male	Female	Age(Mean)
**Cases**	53.6%	46.4%	23.29 months

**Controls**	53.6%	46.4%	23.28 months

Haemoglobin was measured at the time of sample collection using a Coulter counter (Beckman Coulter Inc, Miami, USA). Stored plasma samples were tested for parvovirus B19 IgM and IgG at the same time using the Biotrin Parvovirus B19 IgM and IgG Enzyme Immunoassay kits (Biotrin International Ltd, Dublin, Ireland). Samples in which red cells were incompletely removed during the collection and storage process were included, otherwise specimens were tested as per the kit manufacturer's instructions. All case-control samples were tested for IgM in duplicate, and the test repeated if the first two results differed in outcome (positive, negative or equivocal). For all further analyses equivocal readings were considered as negative. DNA was extracted from 50 μl of the selected plasma samples into 200 μl final volume using the ABI6100 system (Applied Biosystems, California). 5 μl aliquots of extracted DNA were then used in 25 μl PCR reactions with IQ SYBR Green supermix (Bio-rad, California). Primer concentration was optimized at 0.3 μmolar of each of primers B19 R and B19 F for detection of *Human parvovirus B19 *genotypes 1-3 based on the NS1 gene [12]. The Real-time reaction was performed in a Bio-rad Chromo 4 cycler. Due to equipment breakdown PCR results could only be obtained for 198 of the matched pairs of samples. Extracted DNA from Parvovirus B19 Standard (NIBSC, UK) was used for positive control and to generate a standard curve for quantitation. Water was used for negative control and melting curve analysis was performed.

### Statistical analyses

In order to account for matching, the case-control data were analysed with McNemar's exact test using StatXact software (Cytel Inc, Cambridge, USA). The relationship between *P. falciparum *parasitaemia and B19 IgM was analysed with Fisher's exact test usingGraphPad software (GraphPad Software, Inc., La Jolla, USA.)

In calculating our sample size we assumed that around 4.5% of the non-severe anaemia studied samples would be B19 IgM or DNA positive. This was thought to be a conservative estimate, being only one quarter of the figures derived from our previous case/control study in PNG. On this basis, a study of 300 cases matched to equal numbers of controls would have 80% power to detect an odds ratio for acute parvovirus infection in cases compared to controls of 2.57 at a confidence level of >0.95. This power calculation was done using SAM 2.1 computer program [13].

This study was approved by the KEMRI/National Ethical Review Committee in Kenya and the University of Sydney Human Research Ethics Committee in Australia, reference 05-2006/3/9118.

## Results

After samples (cases or controls) that were insufficient or missing data were excluded, 264 matched pairs were tested for the presence of B19 IgM. 198 of these matched pairs were tested for erythrovirus DNA and 271 cases and 265 controls were also tested for B19 IgG.

**Figure 1:**
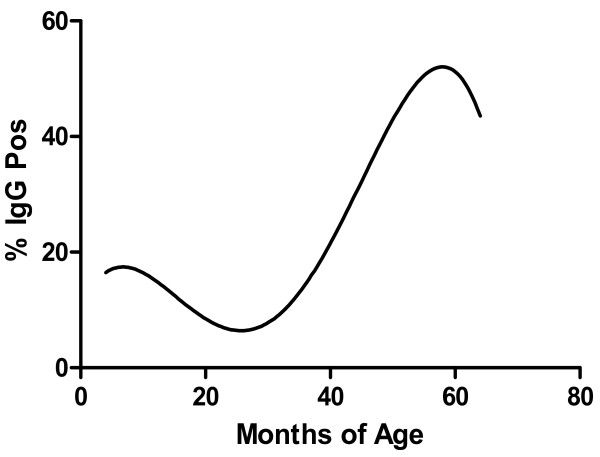
**Percentage of Kenyan children with B19 IgG by age (Polynomial 4^th ^order regression curve)**.

The frequency of B19 IgM positive samples in children with severe anaemia was 10/264 (3.7%) compared to 5/264 (1.9%) in the controls. This difference was not statistically significant, odds ratio (OR) = 2.00 (CI_95 _[0.62, 6.06], McNemar's exact test p = 0.3018. Prevalence of B19 IgG increased with age (Table 2) (Figure 1). There was no significant difference between matched cases and controls in B19 IgG (35 (14.8%) vs 32 (13.6%)), OR = 1.103 (CI_95 _[0.66, 1.89], McNemar's exact test, p = 0.7982), or the detection of B19 DNA (6 (3.0%) vs 5 (2.5%)), OR = 1.2 (CI_95 _[0.33, 4.01], McNemar's exact p = 1) (Table 3). Of the samples from the matched pairs that were PCR positive, the quantitative PCR results (International Units/reaction) for cases were 201, 5342, 6133, 9954, 1.8 × 10^4^, 3 × 10^4^; and for controls 32, 2071, 2520, 4384 and 2.48 × 10^4 ^(Table 4).

**Table 2:** IgM and IgG positivity by age in children admitted to Kilifi district hospital

Months of age	IgM positive			IgG positive		
	**Cases**	**Controls**	**Overall**	**Cases**	**Controls**	**Overall**

**0-6**	1/47 (2.1%)	0/40 (0%)	**1/87** **(1.1%)**	7/43 (16.3%)	10/37 (27.0%)	**17/80** **(21.3%)**

**7-12**	1/62 (1.6%)	0/68 (0%)	**1/130** **(0.8%)**	6/57 (10.5%)	2/57 (3.5%)	**8/114** **(7.0%)**

**13-18**	0/47 (0%)	2/43 (4.7%)	**2/90** **(2.2%)**	2/39 (5.1%)	5/38 (13.2%)	**7/77** **(9.0%)**

**19-24**	2/37 (5.4%)	1/32 (3.1%)	**3/69** **(4.3%)**	6/29 (20.7%)	2/26 (7.7%)	**8/55** **(14.5%)**

**25-30**	2/36 (5.6%)	0/40 (0%)	**2/76** **(2.6%)**	6/29 (20.7%)	3/34 (8.8%)	**9/63** **(14.3%)**

**31-36**	1/21 (4.8%)	0/20 (0%)	**1/41** **(2.4%)**	1/20 (5.0%)	1/19 (5.3%)	**2/39** **(5.1%)**

**37-42**	2/17 (11.8%)	0/20 (0%)	**2/37** **(5.4%)**	4/17 (23.5%)	5/19 (26.3%)	**9/36** **(25%)**

**43-48**	1/16 (6.3%)	2/12 (16.7%)	**3/28** **(10.7%)**	3/15 (20%)	2/12 (16.7%)	**5/27** **(18.5%)**

**49-54**	0/7 (0%)	0/9 (0%)	**0/16** **(0%)**	0/6 (0%)	4/7 (57.1%)	**4/13** **(30.8%)**

**55-60**	0/6 (0%)	1/5 (20%)	**1/11** **(9.1%)**	2/5 (40%)	2/5 (40%)	**4/10** **(40%)**

**61-66**	1/12 (8.3%)	0/10 (0%)	**1/22** **(4.5%)**	4/12 (33.3%)	1/9 (11.1%)	**5/21** **(23.8%)**

**Total**	**11/308** **(3.6%)**	**6/299** **(2.0%)**	**17/607** **(2.8%)**	**41/272** **(15.1%)**	**37/263** **(14.1%)**	**78/535** **(14.6%)**

Data.from samples collected in 1999-2004, including unpaired samples.

**Table 3:** Association of B19 IgM and PCR positivity among cases (having severe anaemia Hb ≤ 50 g/l) as compared to controls.

		IgM		IgM > 5 × COV		PCR		IgM+PCR*	
		**Controls**		**Controls**		**Controls**		**Controls**	

		+	-	+	-	+	-	+	-

**Cases** (Hb ≤ 50 g/l)	+	0	10	0	7	0	6	0	3
	
	**-**	5	249	0	257	5	187	1	194

Exact Odds Ratio		2.00 CI_95 _[0.62, 6.06]		+ Inf CI_95 _[1.65, +Inf]		1.2 CI_95 _[0.33, 4.01]		3.00 CI_95 _[0.33, 77.48]	

Exact McNemar test 2-sided p		p = 0.3018		p = 0.01563		p = 1		p = 0.625	

Numbers refer to matched case-control control pairs.

* only double positive sample scored as positive.

**Table 4:** Data from the 429 samples from among the matched pairs on which all three assays (B19 IgM, IgG and DNA) were performed

Cases	PCR Positive Number (IU/Reaction)	PCR Negative
B19 IgM-, IgG-	3 (201, 6133, 9954)	182

B19 IgM +, IgG-	1 (1.8 × 10^4^)	1

B19 IgM +, IgG+	2 (3 × 10^4^, 5342)	1

B19 IgM-, IgG+	0	28

**Controls**		

B19 IgM-, IgG-	4 (2.48 × 10^4^, 2071, 2520, 4384)	177

B19 IgM+, IgG-	0	3

B19 IgM+, IgG+	1 (32)	1

B19 IgM-, IgG+	0	26

In B19 IgM testing the highest Optical Density values (i.e. Optical Density > 5 × cut-off OD) were all (n = 7) found among the cases, with none (n = 0) among the controls. The association between these high IgMs and severe anaemia was statistically significant with McNemar's exact two-tailed p = 0.0153. The odds ratio for this effect is inestimable as the numbers of controls with high IgM OD = 0.

Among the matched pairs in the study, 5 cases and 2 controls had a diagnosis of sickle cell disease. One of these (a control) was B19 IgM positive. None of the children with sickle cell disease were B19 PCR positive. Results of blood slide microscopy for malaria parasites were available for 256 of the matched pairs. Of these there were 123 discordant pairs: 85 (69.1%) pairs where the case was positive for *P falciparum *but the control was not, and 38 (30.9%) pairs where the control was positive for *P falciparum *but the case was not. Thus *P falciparum *was significantly associated with severe anaemia (OR = 2.24 (CI_95 _[1.52, 3.37] McNemar's exact test p = 0.0000136)).

*P. falciparum *parasitaemia was not significantly associated with B19 IgM in either the cases or controls (Fisher's exact test 2-tailed p = 0.7296 vs p = .0.6826). There was no significant association between severe anaemia and dual (both B19 IgM and *P falciparum) *positive samples (6 (2.3%) cases as compared with 3 (1.2%) controls. Odds ratio = 2.00(CI_95 _[0.43, 9.23] McNemar's exact test p = 0.5078).

**Figure 2:**
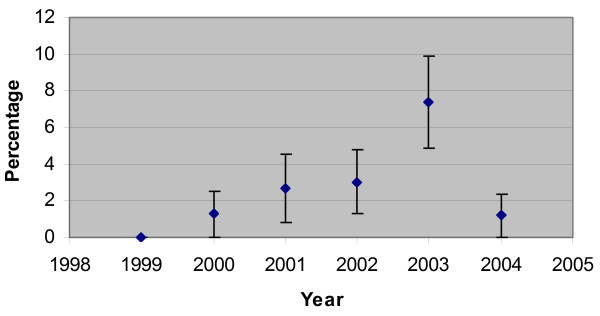
**Percentage of children with B19 IgM in each year tested**. Error bars indicate 95% confidence interval.

The incidence of B19 infection varied between years, with an increase of positive B19 IgM results in the year 2003. In matched pairs from that year 7 (13.0%) of severely anaemic children tested were positive for B19 IgM. 6 of the 7 high IgM results occurred in 2003 (Figure 2).

## Discussion

In contrast to our previous study in PNG among children with clinical malaria, where 30.2% of children with severe anaemia and 12.4% of controls tested positive for B19 IgM, only a small proportion of these Kenyan samples tested were positive for B19 IgM (3.7% of cases and 1.9% of controls). This lower rate of infection in Kilifi is also demonstrated in the IgG results, which show that of these Kenyan children ≤ 6 years only 14.2% have previously had a B19 infection as compared to 63% of children ≤ 5 years in Wosera district, PNG. Possible reasons include differences in climate, socio - economic factors and genetic factors. Furthermore the differences in the type of samples used should also be taken into account. In this study the full range of hospital admissions included all seriously ill children with a range of clinical problems, while in PNG samples came from children with 'clinical malaria' defined as fever with no obvious respiratory cause. It is likely that the selection of children with a history of fever increased the number of acute B19 infections in the PNG samples.

The lower than expected frequency of infections among the present study population meant that the analysis did not have the power to find a significant difference between IgM positive rates in cases and controls with the found odds ratio of 2. Thus, while there are more positives among cases for overall IgM+ and PCR+ these differences did not reach statistical significance. However in spite of the low numbers of B19 infections, a high B19 IgM level (Optical Density> 5 × cut-off Value) is significantly associated with severe anaemia. High IgM levels are most likely to be measured around the time of haemoglobin reduction, and therefore have the strongest association with severe anaemia. This association suggests that in other places where B19 infections are more common among young children (e.g. Eritrea) [14], the contribution of B19 to severe anaemia would be greater. Also, as the incidence of B19 infections fluctuates over time, the cluster of B19 infections in 2003 reveals the potential impact of B19 as an intermittent cause of severe anaemia in this area of sub-Saharan Africa. Further studies are required to estimate the frequency of B19 infections among young children in areas where severe anaemia is a major problem.

In the current study *P. falciparum *infection is shown to be a cause of severe anaemia in this area, as has been documented previously [2], however we did not find a significant association between the prevalence of B19 IgM positivity and *P. Falciparum *parasitaemia in children presenting with severe anaemia.

## Conclusions

High B19 IgM levels were significantly associated with severe anaemia, being found only among the cases. This suggests that at least 2.7% of cases of severe anaemia in the population of children admitted to Kilifi District Hospital were precipitated by B19. While this is a relatively small proportion, this has to be evaluated in the light of the IgG data which shows that less than 15% of children in the study had contracted B19, a figure much lower than reported in other tropical areas.

## Competing interests

Biotrin International Ltd donated the Parvovirus B19 EIA kits, otherwise the authors declare no competing interests.

## Authors' contributions

JW, NG and JT performed the nucleic acid amplification and quantitation. JW and NG performed the serology. JW, YC, NP, TW and CN participated in research design, data analysis and drafting of the manuscript. All authors read and approved the final manuscript.

## Pre-publication history

The pre-publication history for this paper can be accessed here:

http://www.biomedcentral.com/1471-2334/10/88/prepub
